# Development of *Agrobacterium*-mediated transient expression system in *Caragana intermedia* and characterization of *CiDREB1C* in stress response

**DOI:** 10.1186/s12870-019-1800-4

**Published:** 2019-06-06

**Authors:** Kun Liu, Qi Yang, Tianrui Yang, Yang Wu, Guangxia Wang, Feiyun Yang, Ruigang Wang, Xiaofei Lin, Guojing Li

**Affiliations:** 1 Key Laboratory of Plant Stress Physiology and Molecular Biology, Inner Mongolia Agricultural University, Hohhot, People’s Republic of China; 2 College of Food Sciences and Engineering, Inner Mongolia Agricultural University, Hohhot, People’s Republic of China; 3 Key Laboratory of Forage and Endemic Crop Biotechnology, Ministry of Education, Inner Mongolia University, Hohhot, People’s Republic of China

**Keywords:** *Caragana intermedia*, Transient expression, *CiDREB1C*, Abiotic stress

## Abstract

**Background:**

The *Agrobacterium*-mediated transient transformation is a versatile and indispensable way of rapid analyzing gene function in plants. Despite this transient expression system has been successfully applied in a number of plant species, it is poorly developed in *Caragana intermedia*.

**Results:**

In this study, we established an *Agrobacterium*-mediated transient expression system in *C. intermedia* leaves and optimized the effect of different *Agrobacterial* strains, several surfactants and the concentration of Silwet L-77, which would affect transient expression efficiency. Among the 5 *Agrobacterial* strains examined, GV3101 produced the highest *GUS* expression level. Besides, higher level of transient expression was observed in plants infiltrated with Silwet L-77 than with Triton X-100 or Tween-20. Silwet L-77 at a concentration of 0.001% greatly improved the level of GUS transient expression. Real-time PCR showed that expression of *CiDREB1C* was highly up-regulated in transiently expressed plants and reached the highest level at the 2nd day after infiltration. Based on this optimized transient transformation method, we characterized *CiDREB1C* function in response to drought, salt and ABA treatment. The results showed that transiently expressed *CiDREB1C* in *C. intermedia* leaves could enhance the survival rate and chlorophyll content, and reduce the lodging rate compared with the control seedlings under drought, salt and ABA treatments. Furthermore, the rate of leaf shedding of *CiDREB1C* transient expression seedlings was lower than that of the control under ABA treatment.

**Conclusions:**

The optimized transient expression condition in *C. intermedia* leaves were infiltrated with *Agrobacterial* strains GV3101 plus Silwet L-77 at a concentration of 0.001% added into the infiltration medium. Transiently expressed *CiDREB1C* enhanced drought, salt and ABA stress tolerance, indicated that it was a suitable and effective tool to determine gene function involved in abiotic stress response in *C. intermedia*.

**Electronic supplementary material:**

The online version of this article (10.1186/s12870-019-1800-4) contains supplementary material, which is available to authorized users.

## Background

*Agrobacterium*-mediated transient transformation is a technique to get transient and high-level expression of target genes, which is facile and versatile for the characterization of gene function in plants, including analysis of gene promoter properties, transcription factor activity, protein subcellular localization and protein-for-protein interactions. In contrast with the stable genetic transformation, one benefit of transgene introduction using Agroinfiltration is that it does not require time consuming screening of transgenic plants, and the results can frequently be got in days, which is pretty suitable for plants that are difficult to develop regeneration systems. Agroinfiltration was initially developed using *Nicotiana benthamiana* [1], and now this method efficiently works in many plant species including rice (*Oryza sativa*) [2], Medicago (*Medicago truncatula*) [3], tomato (*Lycopersicon esculentum*) [4], cocoa (*Theobroma cacao*) [5], grapevine (*Vitis vinifera*) [6], *Arabidopsis thaliana* [7], soybean (*Glycine max*) [8], wheat (*Triticum monococcum*) [9], tamarisk (*Tamarix hispida*) [10] and birch (*Betula platyphylla*) [10].

At present, the main methods of transient expression in plants include *Agrobacterium*-mediated infiltration, gene gun, mesophyll-protoplasts PEG-mediated or electroporation transfection and plant viral vector-mediated method [11]. To date, the development of *Agrobacterium*-mediated transient expression system in legume species has been reported to be quite limited and most of the study mainly focused on soybean, pea, Medicago and lotus. King et al. [8] has developed and optimized the Agroinfiltration of soybean, and the results showed that *Agrobacterium*-mediated *GUS* reporter gene expression level is increased with vacuum infiltration followed by sonicating, whereas the transient transformation efficiency is not sufficient. In addition, the soybean leaf tissue shows minimal response to syringe infiltration and histochemical GUS staining in syringe-infiltrated soybean leaves, it was inconsistent and occurred in only a small fraction of leaf tissue [8]. Nanjareddy et al. [12] also used the same method to optimize the transient expression system of *Phaseolus vulgaris*, and 60%~ 85% of cells on the leaf surface showed GUS staining after infiltrating with suspension containing acetosyringone (a phenolic compound that can attract *Agrobacterial* cells to the wounded plant tissues via chemotaxis and induces the *Vir* genes to initiate T-DNA transfer [13]) and Silwet L-77, while the procedure is sophisticated and time-consuming. In order to improve the transient transformation efficiency of *Lotus japonicus*, Kimura et al. [14] has screened out a phenolic compound, Chloroxynil, among a variety of compounds, which is able to increase the efficiency of transient transformation 6 times higher than that of acetosyringone. However, Chloroxynil is expensive and highly toxic.

*Caragana intermedia*, a leguminous deciduous perennial shrub, is widely distributed at arid and semi-arid desert areas in Shanxi Province, Inner Mongolia and Ningxia Autonomous Regions of China. It has properties in tolerance of cold, drought, salinity and barren conditions, plays essential roles in sand fixation, broad adaptability to desert area, and with high forage value. Thorough study of the stress tolerance molecular mechanism and exploiting the resources of stress-resistance genes in *C. intermedia* would provide new insights and lay the foundation for genetic engineering and molecular improvement of stress resistance in agricultural and forestry crops. The genome of the *C. intermedia* has not been sequenced yet, but it is not an impossible task to screen resistance gene from the transcriptome database and characterize genes function. Due to lack of stable genetic transformation system of *C. intermedia*, it is urgent to develop a transient transformation system, so that the function of unknown gene could be studied through transient expression system quickly and efficiently. The *dehydration responsive element binding protein 1C* (*DREB1C*) gene plays a critical role in responding to cold stress in plant. *AtDREB1C*, also known as *C-repeat binding factor 2* (*CBF2*), encodes a type of transcription factor that could recognize specifically to the C-repeat (CRT)/dehydration response element (DRE) present in the promoter region of a set of stress-related genes, including *CORs* (cold-regulated) and *RDs* (response to desiccation) [15]. *DREB1C* has been isolated in many plant species, such as rice, tobacco (*Nicotiana tabacum*), maize (*Zea mays*), grape (*Muscadinia rotundifolia*), pepper (*Capsicum annuum*) and cassava (*Manihot esculenta*) [16–19], suggesting that DREB1C is quite conserved in plants. Overexpression of *DREB1C* in *Arabidopsis* resulted in growth retardation and dwarf phenotype, whereas it would also lead up to the enhancement of tolerance to abiotic stress such as cold, drought and dark or hormone induced leaf senescence. The function of *CiDREB1C* has recently been conformed in Arabidopsis [20].

In this study, we developed an efficient transient expression system mediated by *Agrobacterium* in *C. intermedia* leaves in order to verify this system in gene function study, we transiently expressed *CiDREB1C* in *C. intermedia* leaves based on this optimized method and characterized its function in response to abiotic stress and ABA treatment.

## Results

### GV3101 was the most suitable strain for the transient expression of *C. intermedia* leaves

**Fig. 1:**
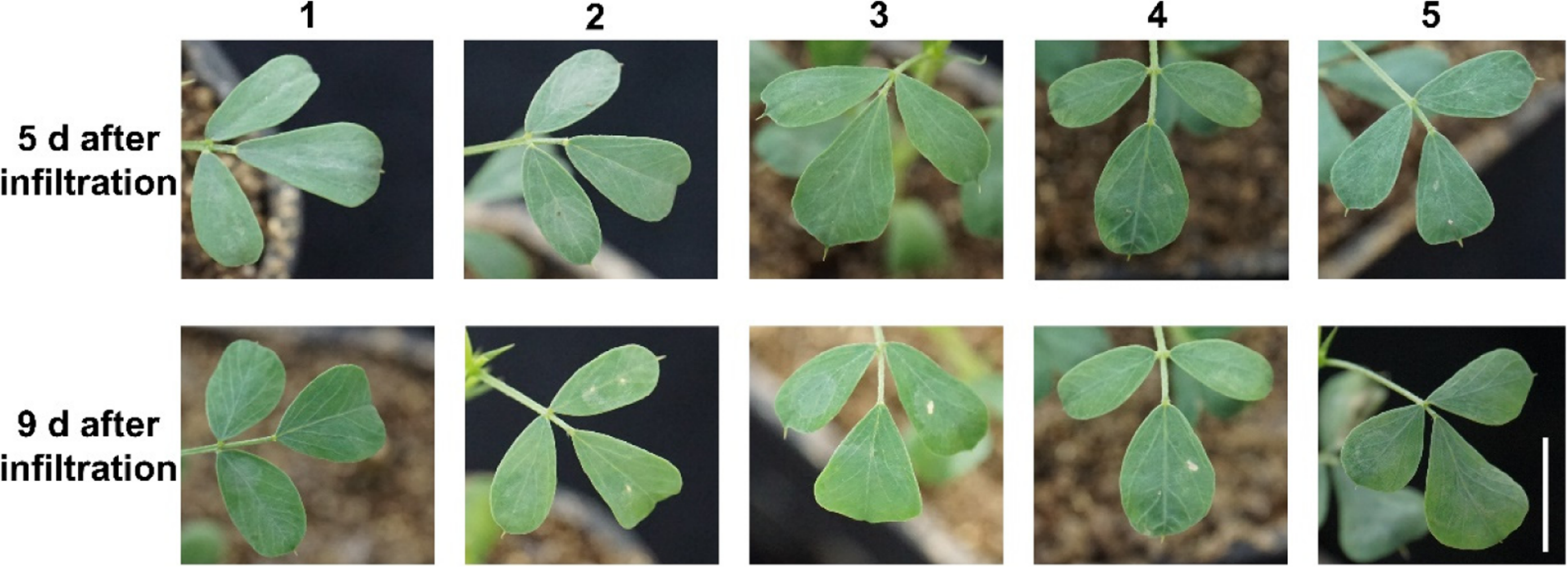
Phenotype of *C. intermedia* leaves after infiltrated with GV3101, EHA105, EHA101, LBA4404 and AGL1 1. Infiltration media + GV3101(pCambia1305.2) + 0.001% Silwet L-77 2. Infiltration media + EHA105(pCambia1305.2) + 0.001% Silwet L-77 3. Infiltration media + EHA101(pCambia1305.2) + 0.001% Silwet L-77 4. Infiltration media + LBA4404(pCambia1305.2) + 0.001% Silwet L-77 5. Infiltration media + AGL1(pCambia1305.2) + 0.001% Silwet L-77 bar = 1 cm

**Fig. 2:**
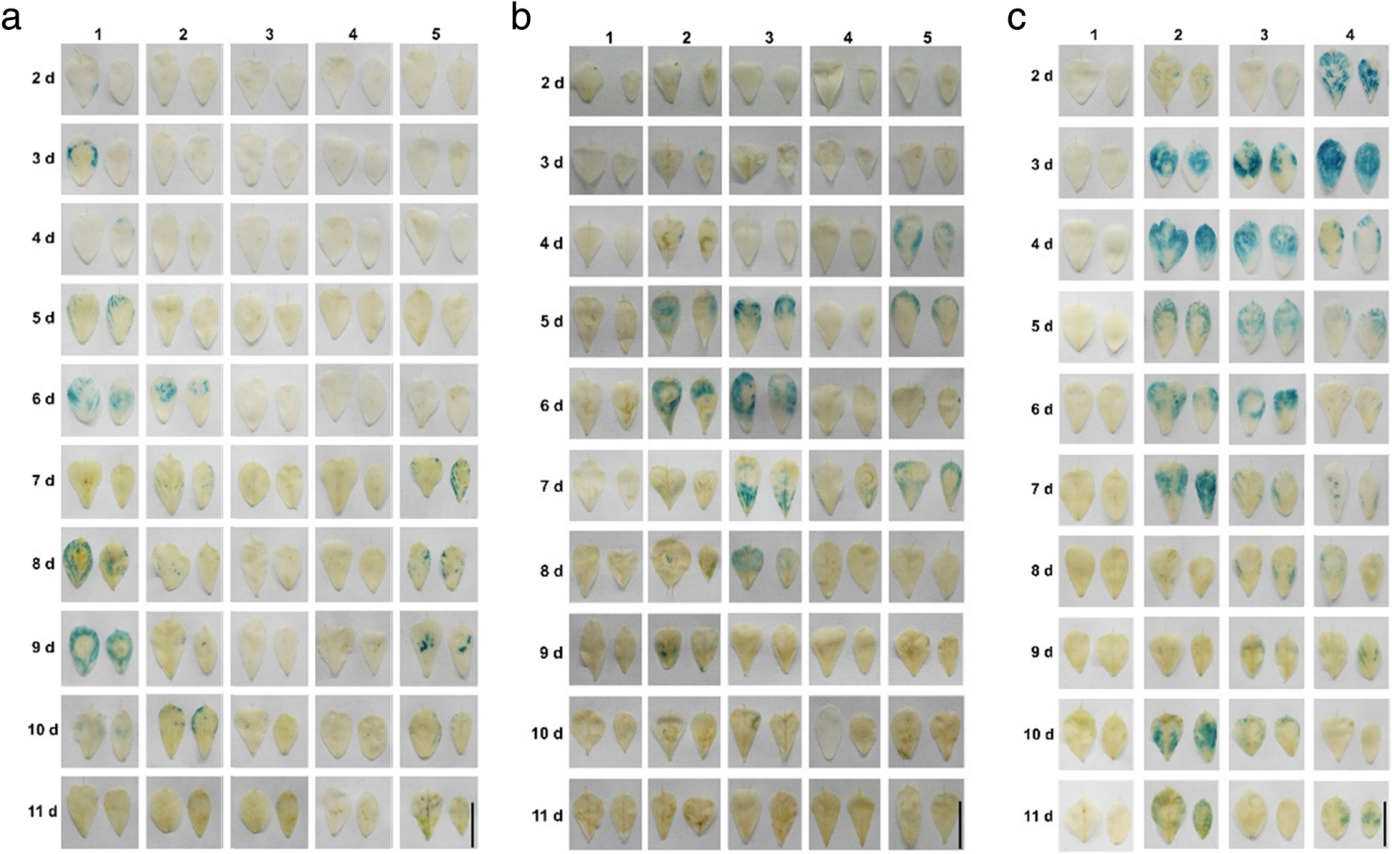
GUS staining analysis of different surfactants, surfactant concentration and *Agrobacterial* strains in transiently expressed *C. intermedia* leaves **a.** 1. Infiltration media + GV3101 (pCambia1305.2) + 0.001% Silwet L-77; 2. Infiltration media + EHA105 (pCambia1305.2) + 0.001% Silwet L-77; 3. Infiltration media + EHA101 (pCambia1305.2) + 0.001% Silwet L-77; 4. Infiltration media + LBA4404(pCambia1305.2) + 0.001% Silwet L-77; 5. Infiltration media + AGL1(pCambia1305.2) + 0.001% Silwet L-77 bar = 1 cm. **b.** 1. Infiltration media + GV3101(negative control); 2. Infiltration media + GV3101 (pCambia1305.2); 3. Infiltration media + GV3101(pCambia1305.2) + 0.01% Silwet L-77; 4. Infiltration media + GV3101(pCambia1305.2) + 0.01% Tween-20; 5. Infiltration media + GV3101(pCambia1305.2) + 0.01% Trion X-100 bar = 1 cm. **c.** 1. Infiltration media + GV3101(negative control); 2. Infiltration media + GV3101(pCambia1305.2) + 0.001% Silwet L-77; 3. Infiltration media + GV3101(pCambia1305.2) + 0.005% Silwet L-77; 4. Infiltration media + GV3101(pCambia1305.2) + 0.01% Silwet L-77 bar = 1 cm

Studies have shown that the infection capability of *A. tumefaciens* to plants is varying, and the transient expression efficiency of plants would be significantly affected by the genetic background of *A. tumefaciens* strains [21]. To test the effect of *A. tumefaciens* strains on the transient gene expression of the *C. intermedia* leaves, we compared five widely used *A. tumefaciens* strains, including GV3101, EHA105, EHA101, LBA4404 and AGL1. Among them, the chromosomal background of EHA101, EHA105 and GV3101 strains are all from C58. While AGL1 strain belongs to C58 and RecA, and LBA4404 belongs to Ach5. Consistently, all of the *A. tumefaciens* strains are resistant to rifampicin. Besides, pTiBo542DT-DNA was also in AGL1, EHA101 and EHA105 strains. The details of five *A. tumefaciens* strains could be found in Table 1. The leaf phenotype was observed (Fig. 1) and the GUS reporter gene expression was detected after infiltration. According to the GUS staining results, *GUS* reporter gene expression was observed in GV3101 strain at the 2nd day after infiltration, and lasted to the 9th day, and the expression level of *GUS* reporter gene was the highest at the 8th and the 9th day (Fig. 2a). However, the leaves infiltrated by EHA105 strain were just stained at the 6th day after injection, and sustained until the 10th day, which show a sporadic punctate distribution on the leaves. On the 7th day after infiltration, the leaves appeared GUS staining at the AGL1 strains infiltration condition, and the staining lasted to the 9th day and reached the highest level. However, the persistent time of GUS staining was brief. None of the leaves were stained with LBA4404 or EHA101 strains under the infiltration condition (Fig. 2a). These results suggested that *A. tumefaciens* strains GV3101 was the most suitable strain for the transient expression of *C. intermedia* leaves. Therefore, we take GV3101 as the candidate strain for the subsequent studies.

**Table 1:** *Agrobacterium* strains used in this study

Strains	Chromosomal background	Resistance gene^a^	Ti plasmids	Opines
AGL1	C58, RecA	rif, carb	pEHA105 (pTiBo542DT-DNA)	Succinamopine
EHA101	C58	rif, kan	pEHA101 (pTiBo542DT-DNA)	Nopaline
EHA105	C58	rif,	pEHA105 (pTiBo542DT-DNA)	Succinamopine
LBA4404	Ach5	rif, strep	pAL4404	Octopine
GV3101	C58	rif, gent	pMP90 (pTiC58DT-DNA)	Nopaline

^a^Resistance genes used to select for chromosomal backgrounds and Ti plasmids. Rif, rifampicin resistance; carb, carbenicillin resistance; kan, kanamycin resistance; gent, gentamycin resistance; strep, streptomycin resistance

### Silwet L-77 is the most effective surfactant for *Agrobacterium-*mediated transient expression in *C. intermedia* leaves

To assay the effect of surfactants on the transient expression efficiency, three different surfactants, Silwet L-77 (0.01%), Triton X-100 (0.01%) and Tween-20 (0.01%) were added to the infiltration solution. GUS staining results showed that there was no significant *GUS* gene expression under different conditions within 3 days after infiltrating. Four days after infiltration, *A. tumefaciens* infiltrated leaves with Triton X-100 were mostly stained at the edges of leaves and lasted until the 7th day (Fig. 2b). After infiltrating for 5~6 days, the infiltrated leaves without any surfactants or with Silwet L-77 had obvious *GUS* reporter gene expression, and the stained area accounted for over 70% of the entire leaf area (Fig. 2b). The expression of *GUS* reporter gene in leaves with Silwet L-77 was still detectable at 7~8 days after infiltration, and the staining area was over 50% of the entire leaf area, while no expression of *GUS* was observed in leaves without any surfactant by then. Furthermore, the leaves infiltrated with Tween-20 showed only slight *GUS* expression on the 7th day (Fig. 2b), and no detectable *GUS* expression observed thereafter. These results suggested that Silwet L-77 is the most effective surfactant for *Agrobacterium-*mediated transient expression in *C. intermedia* leaves and the *GUS* reporter gene expressed to the highest level at 5~7 days after infiltration.

To investigate the effect of surfactant concentration on transient expression efficiency of *C. intermedia*, we select Silwet L-77 as the suitable surfactant based on the above results. According to the results of GUS staining (Fig. 2c), the *GUS* reporter gene began to express under 0.01% Silwet L-77 at the 2nd day, which was rapid, and lasted from the 2nd day to the 5th day, the expression level on the 3rd day was the highest and all the leaves were basically stained, but the duration of staining was short. With 0.005% Silwet L-77, the GUS staining lasted from the 3rd to the 6th day after infiltration, and edges as well as partial of the leaf blades were staining, which covers about 50% of the entire leaf area (Fig. 2c). However, the staining duration was still transient. Under 0.001% Silwet L-77, the response of *GUS* gene in the leaves was rapid, which was detected on the 2nd day after infiltration and reached the highest expression level on the 4th day with staining area basically distributed throughout the entire leaf. The *GUS* gene expression lasted until the 7th day with a high level. Interestingly, the GUS staining was still detectable on the 10th or the 11th day after infiltration (Fig. 2c). In conclusion, addition of 0.001% Silwet L-77 in the media has the highest *GUS* reporter gene expression efficiency among all the concentrations tested. Therefore, we used this infiltration condition for further research.

Although there were still other key factors such as concentration of acetosyringone, *Agrobacterium* and MgCl_2_, according to the literatures so far, some scholars had reported that higher transient expression level was obtained with 100 μmol /L acetosyringone, 10 mmol/L MgCl_2_ and 0.7~0.8 OD_600_ of *Agrobacterium* [8, 12, 22]. Thus, we did not explore and optimize these conditions in this paper.

### Transient expression of *CiDREB1C* enhanced tolerance to drought and salt stress in *C. intermedia*

**Fig. 3:**
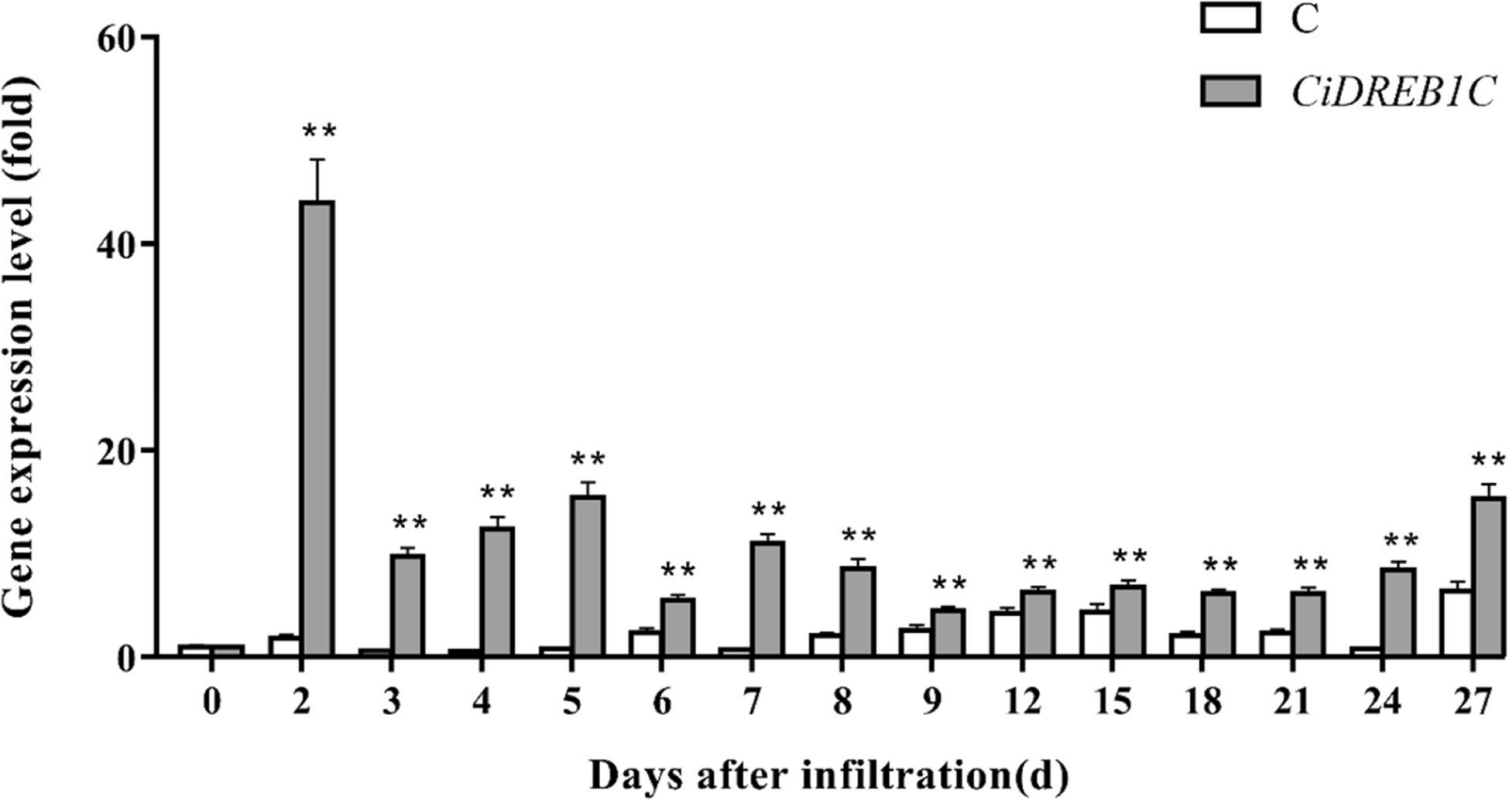
Quantification analysis of the transiently expressed *CiDREB1C* in *C. intermedia* leaves by RT-PCR The *CiDREB1C* gene expression level in leaves of *C. intermedia* after infiltrating with pCanG-HA empty vector and *CiDREB1C* was detected by relative quantitative real-time PCR. The Expression values were calculated using the 2^-ΔΔCT^ method and *CiEF1α* was used as reference gene. The error bars represent the means of three technical replicates of each biological replicate ± SD. The experiments were performed two independent biological replicates with similar result. Statistical significance differences from control were determined by Student’s *t* test (**P < 0.01). The column was made by Graphpad prism 7. C: control

To test the applicability of our optimized transient expression system, the well-studied *DREB1C* was cloned from *C. intermedia*, and its function in drought and salt resistance has been proved in transgenic Arabidopsis [20]. Using quantitative real-time PCR, we detected the expression level of *CiDREB1C* in leaves of *C. intermedia* after infiltration. The results showed that the expression of *CiDREB1C* reached the highest level at the 2nd day after transient expression, and the expression level was about 44~45 times of 0 day. Then it decreased gradually but still be detectable on the 27th day (Fig. 3). The Expression level of *CiDREB1C* in transient expression seedlings was significantly higher than that of the control (*P* < 0.01).

**Fig. 4:**
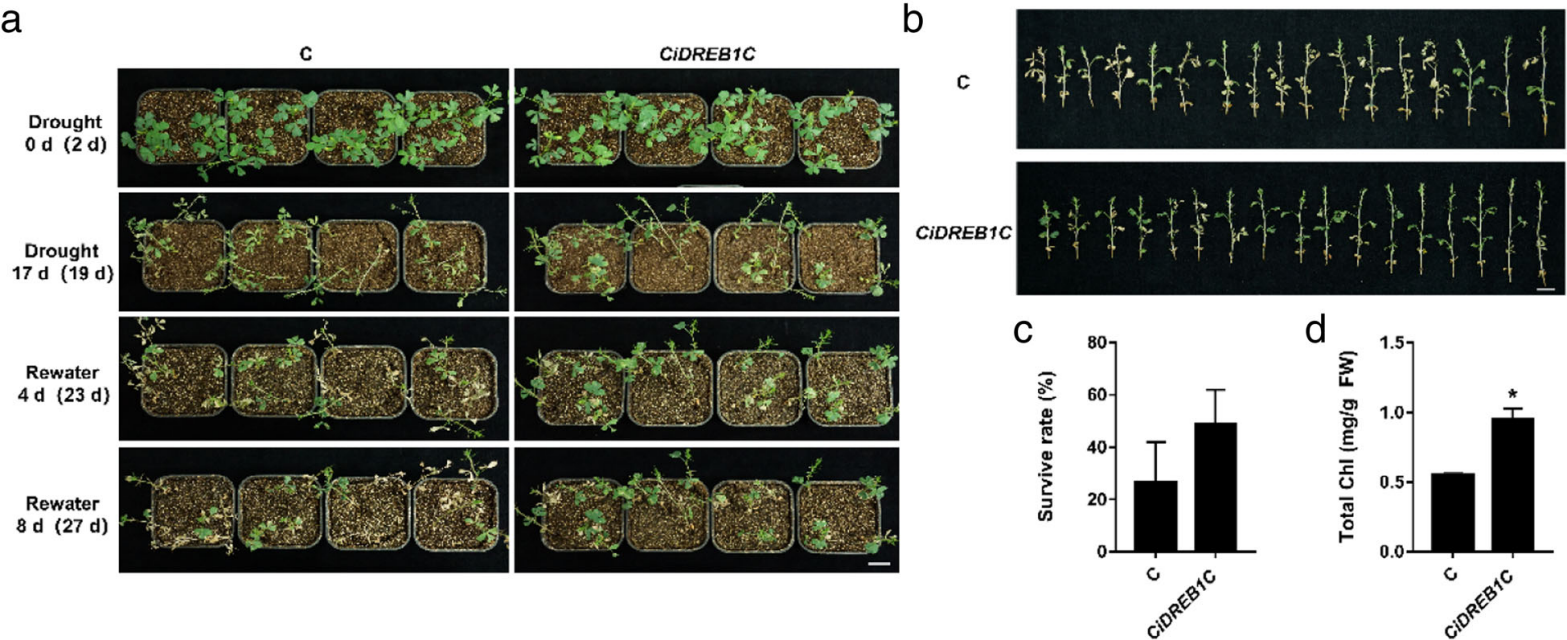
Drought resistance detection of *CiDREB1C* transiently expressed *C. intermedia* seedlings **a.** Plain view of *CiDREB1C* transiently expressed *C. intermedia* seedlings grown in soil under drought treatment. Bar = 2 cm *n* = 16. **b.** The detached seedlings of both the control (up panel) and the *CiDREB1C* transgenic *C. intermedia* (lower panel) after re-watered for 8 days. Bar = 2 cm n = 16. **c.** The survival rate of both the control and the *CiDREB1C* transgenic *C. intermedia* seedlings after re-watered for 8 days. **d.** The chlorophyll content of both the control and the *CiDREB1C* transgenic *C. intermedia* seedlings after re-watered for 8 days. Days after infiltration was indicated in parentheses. Statistical significance differences from control were determined by Student’s *t* test (*P < 0.05). The column was made by Graphpad prism 7. C: control

To confirm the role of *CiDREB1C* in response to abiotic stresses, the *CiDREB1C* transiently expressed *C. intermedia* seedlings was exposed to drought and high salt stress treatment at 2 or 3 days after infiltrating. The growth of seedlings is uniform before drought treatment (Fig. 4a, Additional file 2: Figure S2)1. After exposing to drought for 17 days, most of the control seedlings (infiltrated empty vector) were wilted and lodged, and leaves showed evidently shedding and yellowish phenotype, whereas the *CiDREB1C* transient expression seedlings (infiltrated with pCanG-HA*-CiDREB1C*) appeared relatively healthy (Fig. 4a, Additional file 2: Fig. S2). After re-watering for 8 days, most of the control seedlings were wilt and dead, and displayed a low survival rate (26.4%), whereas the transgenic seedlings had a higher survival rate (48.9%) (Fig. 4b and c, Additional file 2: FigureS2). The total chlorophyll content of *CiDREB1C* transient expression seedlings was 0.94 mg/g FW, significantly higher than that of the control (0.55 mg/g FW) (*P* < 0.05) (Fig. 4d). These data indicated that the *CiDREB1C* enhanced tolerance of transgenic *C. intermedia* to drought stress.

**Fig. 5:**
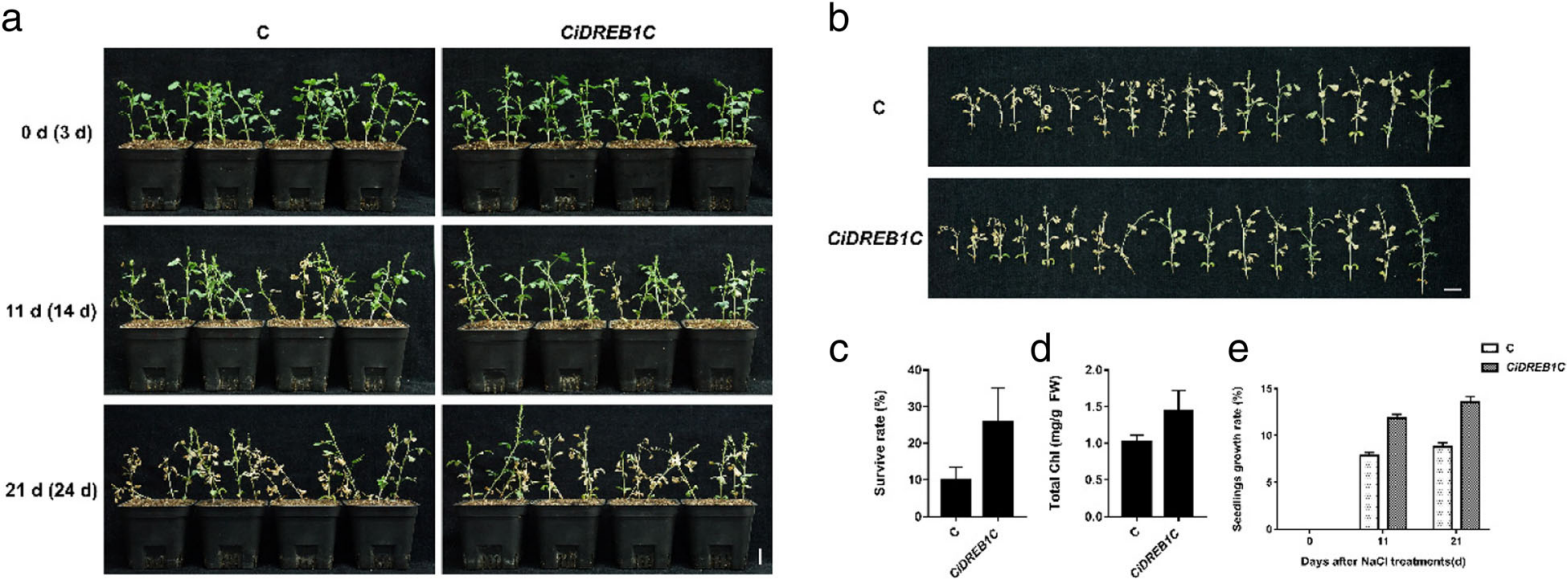
Salt resistance detection of *CiDREB1C* transiently expressed *C. intermedia* seedlings. **a.** Plain view of *CiDREB1C* transiently expressed *C. intermedia* seedlings grown in soil under salt treatment. Bar = 2 cm n = 16. **b.** The detached seedlings of both the control (up panel) and the *CiDREB1C* transgenic *C. intermedia* (lower panel) after 22 days of salt treatment. **c.** The survival rate of both the control and the *CiDREB1C* transgenic *C. intermedia* seedlings after 22 days of salt treatment. **d.** The chlorophyll content of both the control and the *CiDREB1C* transgenic *C. intermedia* seedlings after 22 days of salt treatment. Days after infiltration was indicated in parentheses. **e.** Seedlings growth rate of both the control and the *CiDREB1C* transgenic *C. intermedia* seedlings. The column was made by Graphpad prism 7. C: control

After 250 mmol/L NaCl treatment for 11 days, both the control and the *CiDREB1C* transient expression seedlings appeared wilting and yellowing symptoms, but there was no significant difference between them (Fig. 5a, Additional file 3: FigureS3). After salt treatment for 21 days, the seedlings of both the control and the *CiDREB1C* transiently expressed *C. intermedia* withered and lodged seriously, whereas the control seedlings were more obvious than those of the *CiDREB1C* transient expression seedlings (Fig. 5a, Additional file 3: Figure S3). Statistics of survival rate on the 21st day showed that it was 25.83% in *CiDREB1C* transient expression seedlings, which was much higher than that of the control (10%) (Fig. 5b and c). The chlorophyll content of *CiDREB1C* transiently expressed leaves (1.45 mg/g FW) was higher than that of the control (1.02 mg/g FW) (Fig. 5d). Furthermore, the growth rate of the control was lower than that of *CiDREB1C* transient expression seedlings during the whole salt treatment period (Fig. 5e). Taken together, these results indicated that transient expressed *CiDREB1C* could increase tolerance of *C. intermedia* to salt stress.

### Transient expression of *CiDREB1C* decreased ABA sensitivity in *C. intermedia*

**Fig. 6:**
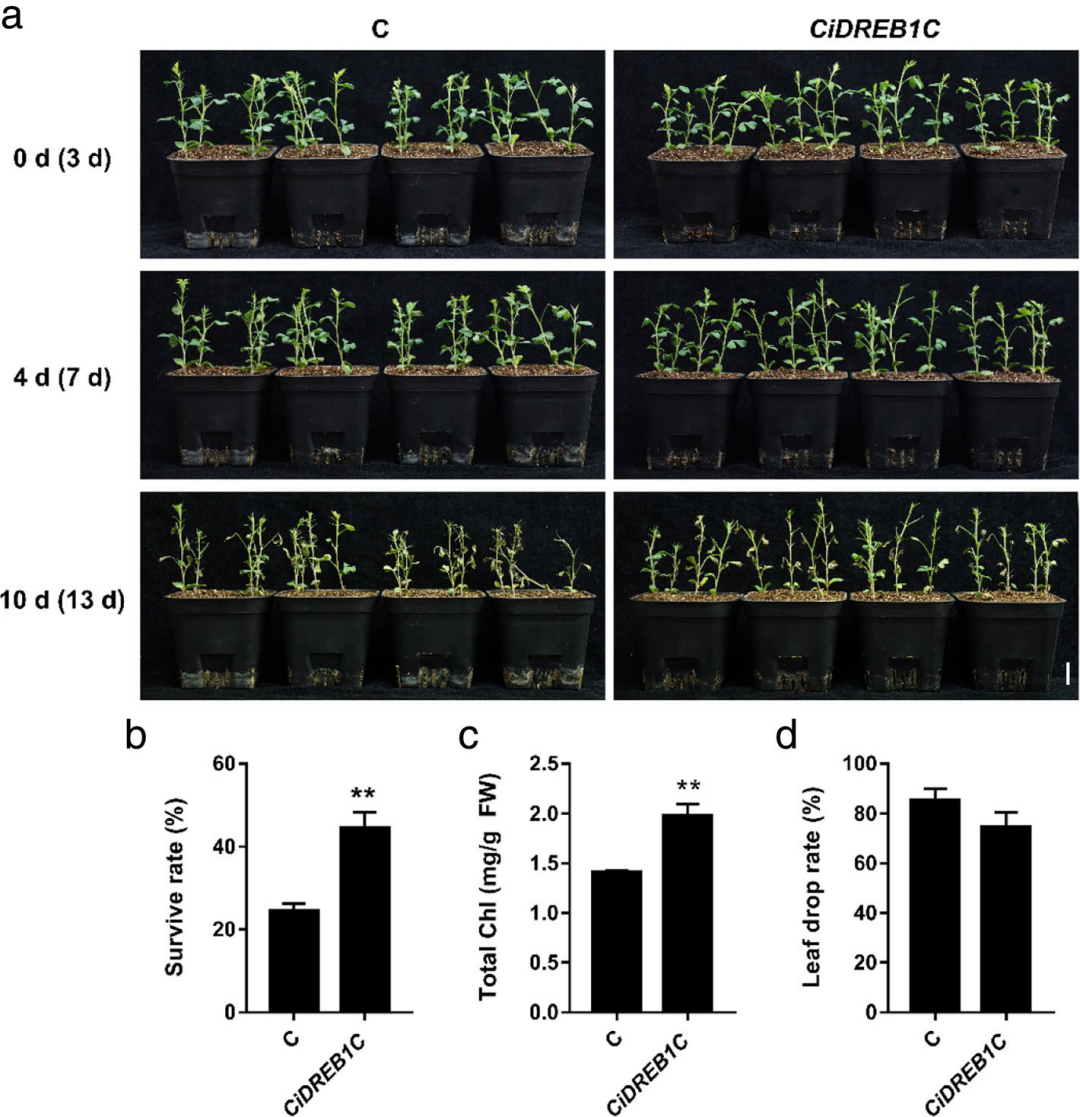
ABA tolerance of *CiDREB1C* transiently expressed *C. intermedia* seedlings. **a.** Plain view of *CiDREB1C* transiently expressed *C. intermedia* seedlings grown in soil under ABA treatment. Bar = 2 cm n = 16. **b.** The survival rate of both the control and the *CiDREB1C* transgenic *C. intermedia* seedlings after ABA treatment for 10 days. **c.** The total chlorophyll content of both the control and the *CiDREB1C* transgenic *C. intermedia* seedlings after ABA treatment for 10 days. **d.** The leaf drop rate of both the control and the *CiDREB1C* transgenic *C. intermedia* seedlings after ABA treatment for 10 days. Days after infiltration was indicated in parentheses. Statistical significance differences from control were determined by Student’s *t* test (**P < 0.01). The column was made by Graphpad prism 7. C: control

Abscisic acid (ABA), an essential phytohormone that could inhibit plant growth, and play a critical role in seed dormancy and germination, leaf maturation, leaf shedding and leaf senescence. To investigate whether transient expression of *CiDREB1C* in *C. intermedia* could affect its sensitivity to ABA, we sprayed ABA aqueous solution uniformly on the seedlings after *Agrobacterium* infiltration for 3 days. The leaves had no significant difference between the control and the *CiDREB1C* transient expression seedlings after spraying ABA solution for 4 days (Fig. 6a, Additional file 4: Figure S4). Ten days after ABA treatment, the control seedlings showed somewhat lodging phenotype (Fig. 6a), while the *CiDREB1C* transient expression seedlings showed no obvious lodging, and had much higher survival rate (44.42%) (*P* < 0.01) (Fig. 6b) and chlorophyll content (1.98 mg/g FW) (P < 0.01) (Fig. 6c) in comparison with the control (24.51% and 1.41 mg/g FW, respectively). In addition, the leaf dropping rate of the control (85.13%) was higher than that of the *CiDREB1C* transient expression seedlings (74.43%) (Fig. 6d). The above results suggest that CiDREB1C plays a positive role in regulating ABA-mediated inhibition of leaf dropping as well as plant growth, which is consistent with the conclusions drew by others [23, 24]. It is also confirmed that the transient expression system is successful to evaluate gene function conveniently in *C. intermedia.*

## Discussion

Plant transient expression is a technique that can achieve overexpression of the target gene in the specific plant in a short period of time, in which the target gene can express within 12 h after the recombinant plasmid enters into the plant cells, and the peak expression level would emerge in 2~4 days [25]. It is a simple, rapid and efficient method for detecting function of the target gene. For species that are difficult to develop genetic transformation systems, the construction of transient expression system is of great significance [26]. There are two main methods for *Agrobacterium*-mediated transient expression: vacuum infiltration and syringe infiltration, and we have tried and compared these two methods. When vacuum infiltration method was used, *C. intermedia* leaves need to be separated from the plants, in addition, the leaves were not entirely infiltrated after sonication and vacuum infiltration. This was potentially caused by the thick stratum corneum and wax coat present on the outermost layer of mature *C. intermedia* leaves. Also, the small leaf blade and the narrow cell gap may be one of the reasons for incapable of fully infiltration. When syringe infiltrated method was used, the living plants could be infiltrated directly without separating the leaves, which was more intuitive for subsequent phenotypic experiment. Moreover, the syringe has small aperture and large thrust, and the suspension of *Agrobacterium* could facially enter into the leaf cells and spread along the whole leaf. Thus, the use of syringe infiltration for *Agrobacterium*-mediated transient expression should be a more desirable method for *C. intermedia*.

*Agrobacterium* strains with different genetic backgrounds have a great influence on the efficiency of transient gene expression [21]. Cao et al. [27] showed that GV2260 had the highest transient expression efficiency compared with EHA105 and LBA4404 in optimizing the transient expression system of spinach. Wu et al. [22] optimized the transient expression system of mulberry trees and found that the mulberry leaves infiltrated by GV3101 strain had higher transient expression efficiency than LBA4404. Andrieu et al. [2] found that the transient expression effect of EHA105 strain was better than AGL1 and LBA4404 in rice leaves, while Mishra et al. [28] optimized the transient expression system of *Withania somnifera* and found that LBA4404 strain had the highest infection efficiency. Up to now, there were many strains reported to be able to infiltrate legumes with high expression efficiency, including GV3101 [14], GV2260 [29], EHA105 [14, 30], LBA4404 [31, 32], AGL1 [3, 33] and J2 [8]. In this study, we compared the genetic background of 5 *Agrobacterium* strains for transient expression in *C. intermedia*, and the GUS staining results revealed that the EHA101 and LBA4404 had no effect on the leaves of *C. intermedia*, and GV3101 strain was more suitable for transient expression system compared to EHA105 and AGL1 (Fig. 2a).

Surfactants are a class of compounds which can reduce the surface tension between water and oil by absorbing at the liquid-liquid interface. According to the different charges, it can be divided into anionic, cationic, amphoteric, and nonionic surfactants. The nonionic surfactants are widely used in the field of molecular biology, because they have less negative effect on plant growth [7]. Usually, the surfactant is amphiphilic and has both hydrophilic and hydrophobic group. Silwet L-77, a type of organosilicone chemistry, has been proved to improve water penetration of hard-to-wet soils and to provide more uniform distribution of applied moisture, and it can improve cuticular penetration of plant surface by spraying. Tween-20, also known as polysorbate-20, is a class of non-ionic surfactant, which is soluble in water and extensively used in biochemical, biomedicine, industry and food field. Triton X-100, a non-ionic detergent, has been most frequently used as a component of cell lysis buffers or other solutions intended to extract and solubilize proteins.

Up to now, there are a number of reports shown that surfactants can improve the efficiency of transient expression. Both Clough et al. [34] and Wu et al. [35] suggested that Silwet L-77 could improve the efficiency of transient expression in Arabidopsis and wheat mediated by *Agrobacterium tumefaciens*, respectively. Nanjareddy et al. [12] showed that 0.001% Silwet L-77 could significantly improve the transient expression efficiency in bean leaves through Sonication assisted Agrobacterium-mediated transformation (SAAT). Ji et al. [26] revealed that 0.01% Tween-20 could definitely increase the transient expression efficiency of many herbaceous plants and trees including *T. hispida*, *B. platyphylla*, *Salix matsudana*, *Aralia mandshurica*, *N. benthamiana* and *A. thaliana*. Zhong et al. [36] added 0.01% Tween-20 into the transient expression system of *Brassica napus* mediated by *Agrobacterium*-mediated root absorption, and attained higher transformation efficiency in comparison with the control. Kim et al. [7] investigated the effects of several surfactants on the transient expression in the *Arabidopsis* leaves and found that 0.01% Triton X-100 and 0.01% Tween-20 both showed the most promotive effects on transient expression in *Arabidopsis* leaves, while the effects of Silwet L-77 were inapparent. In the present study, we selected three commonly used surfactants, Silwet L-77, Tween-20 and Triton X-100 and our results suggested that the Silwet L-77 was the most effective surfactant in the *C. intermedia* transient expression system (Fig. 2b).

Additionally, the concentration of surfactant also affects the efficiency of transient expression. Chen et al. [37] optimized the transient expression system of switchgrass (*Panicum virgatum*) and found that the efficiency of transient expression was affected by the concentration of surfactant. When 0.02% Li700 or Silwet L-77 was added into the system, the efficiency of transient expression was much higher than other concentration. The expression of *GUS* reporter gene, however, would be inhibited to a great extent when the concentration was more than 0.1%. Previously, some scholars [7] optimized the transient expression system of *A. thaliana* and compared the effects of Silwet L-77, Tween-20 and Triton X-100 at different concentrations on the transient expression efficiency. The results implied that compared with other concentrations, the 0.01% Triton X-100 or 0.01% Tween-20 could both significantly improve the transient expression efficiency of *A. thaliana* and the effect of 0.01% Tween-20 is better. While the effects of Silwet L-77 are relatively lower than those of Tween-20 and Triton X-100. When surfactants are applied at a concentration of 0.01% or lower, the transient expression level is conspicuously promoted, yet, when the concentration is higher than 0.05%, it would lead to leaf wilting, yellowing, and seriously weaken the expression level of target gene [7]. Here, we optimized the concentration of Silwet L-77 and the results suggested that the levels of transient expression of 0.001% Silwet L-77 in *C. intermedia* were higher than 0.005 and 0.01% (Fig. 2c). Besides, we also tried to use 0.05% Silwet L-77 to infiltrate the leaves, but it was hard to infiltrate and the possible reason was that the high concentration of Silwet L-77 lead to a higher viscosity of the *Agrobacterial* cells suspensions.

At present, it is not new to study the function of genes in plant through transient expression, and the most widely used species is *N. benthamiana*. Many of the scholars had transiently expressed the target gene in the intact leaves of *N. benthamiana* and performed a series of physiological and biochemical detection, and obtained ideal results [38, 39]. However, considering the limitations of heterologous gene expression, some researchers transiently expressed the target gene in homologous plant and studied the function of the target gene. Ji et al. [26] suggested that *ThbZIP1* was induced by NaCl, PEG6000, NaHCO_3_ and CdCl_2_. When transiently expressed *ThbZIP1* in *T. hispida*, the salt tolerance of transgenic plant significantly enhanced. Moreover, the activities of SOD and POD were markedly increased in the transient expression *T. hispida*, and several *SOD* and *POD* genes were also significantly up-regulated. We used the optimized system to transiently express *CiDREB1C* in *C. intermedia* and detected its resistance to stresses, the results showed that *C. intermedia* seedlings with transient expression of *CiDREB1C* had lower lodging rate, higher survival rate and chlorophyll content compared with the control lines under drought, salt and ABA treatment (Figs. 4 and 6), besides, after ABA treatment, the leaf drop rate of *CiDREB1C* transient expression seedlings was lower than that of the control (Fig. 6d). These results suggested that this transient transformation system was a powerful and effective method allowing the accurate analysis of the gene function in response to abiotic stresses in *C. intermedia*.

## Conclusions

In this study, we developed the transient expression system in *C. intermedia* and optimized the effect of *Agrobacterial* strains, surfactants and concentration of surfactant. Transient expression of *CiDREB1C* in *C. intermedia* resulted in tolerance to drought, salt and ABA stress, compared with the control seedlings. These results will be helpful for understanding the involvement of *DREB1C* in stress resistance and function analysis of other genes in plants that are difficult to develop regeneration systems. This system can also be used to analyze promoter activitiy, TF actions and protein-protein interactions. It provided a new option for gene function investigation in *C. intermedia*.

## Methods

### Plant materials and growth conditions

*C. intermedia* seeds were collected from Helin County, Hohhot, Inner Mongolia, China. No specific field permissions were required to collect the plant samples and seeds. The full, intact and healthy seeds were selected and routinely grown in a 1:3 mixture of peat soil and vermiculite in a greenhouse (22 °C, 16 h light/8 h dark). For transient expression assays, fully expended leaves of three-week-old seedlings were chosen for injection (Additional file 1: Figure S1a).

### *Agrobacterium* strains and plasmids

The *Agrobacterium tumefaciens* strains, GV3101, EHA105, EHA101, LBA4404 and AGL1 were purchased from Shanghai Weidi Biotechnology Co, Ltd. The detailed information of all the five *Agrobacterium* strains were listed in Table 1. The pCAMBIA1305.2 binary vector (kept by the Key Laboratory of Plant Stress Physiology and Molecular Biology, Inner Mongolia Agricultural University) which contains the CaMV35S promoter, *GUSPlus* reporter gene, kanamycin resistance gene for bacteria selection and hygromycin resistance gene for plant selection, was transformed into five *Agrobacterial* strains by electroporation, respectively. The expression of both *GUSPlus* reporter gene and kanamycin resistance gene were driven by CaMV35S promoter and the *GUSPlus* reported gene expressed only in plant cells but not in bacteria cells.

### *Agrobacterium* cell culture and preparation of infiltration suspension

A single colony of *A. tumefaciens* strains GV3101, EHA105, EHA101, LBA4404 and AGL1 harboring pCAMBIA1305.2 binary vector was inoculated in 4 mL Luria Broth (LB) liquid medium (50 μg/mL kanamycin and 25 μg/mL gentamicin for GV3101, 50 μg/mL kanamycin and 20 μg /mL rifampicin for EHA105, EHA101, LBA4404 and AGL1), and was incubated overnight at 28 °C at 200 rpm. On the next day, an aliquot of 1.5 mL *Agrobacterial* cells were transferred to 25 mL fresh LB liquid medium (1:25 ratio, v/v) supplemented with kanamycin (50 μg /mL) and gentamicin (25 μg /mL) for GV3101, or kanamycin (50 μg /mL) and rifampicin (20 μg /mL) for other trains. All the strains were allowed to grow until the cell density reached an OD_600_ of 1.3. The cells were centrifuged at 5000 ~ 6000 rpm for 10 min at 4 °C and the supernatant was discard. The cells were resuspended with the infiltration medium (1/2MS medium (PH5.8), 10 mmol/L MES, 10 mmol/L MgCl_2_, 100 μmol /L acetosyringone) to OD_600_ = 0.7~0.8. To optimize the infiltration condition, several surfactants such as Silwet L-77, Tween-20 and Triton X-100 were added to the infiltration medium, and various concentrations with 0.001% (v/v), 0.005% (v/v) or 0.01% (v/v) of the surfactant were optimized based on the optimal surfactant. Prior to infiltration, the *Agrobacterial* cells suspensions were incubated at room temperature under darkness for 3~4 h since the *Vir* gene in *Agrobacterial* cells need to be fully induced by acetosyringone, and during this time, the *Agrobacterial* cells suspensions should be mixed upside down every half hour since the *Agrobacterial* cells could be uniformly suspended in the 1/2 MS medium.

### Infiltration of the *C. intermedia* leaves

The 3-week-old seedlings was chosen to infiltrate (We chose 3-week-old plants because the leaves of *C. intermedia* were relatively young and fully expanded after growing for about three weeks, which are pretty beneficial for transient expression in *C. intermedia*.). The *Agrobacterial* cells suspensions was pressure-infiltrated to the abaxial surface of *C. intermedia* leaves using a 1 mL disposable needleless syringe (Additional file 1: Figure S1b). Infiltrating leaves in sections until the whole area appears translucent, and leaves were saturated with *Agrobacterial* cells suspensions should be separated and should not touch each other (Additional file 1: Figure S1c). Immediately after infiltration, the plant was covered with dark plastic film for 24 h to maintain a high relative humidity (> 80%), and was kept in dark condition for another 2 days, then the plants were grown in the normal condition (22 °C, 16 h light/8 h dark). Last but not the least, it is necessary to spray ddH_2_O to the plants in the growth chamber, by doing this, the relative humidity of plants surrounding was high and the efficiency of transient expression was improved [7]. The untransformed *C. intermedia* seedlings were used as the negative control.

### β-Glucuronidase (GUS) histochemical staining

The *C. intermedia* leaves were collected in 2~11 days after infiltration and 10~12 leaves from each experimental condition were taken each day. Histochemical GUS staining was carried out as described previously [40]. The infiltrated leaves were submerged in the GUS staining solution (50 mM NaH_2_PO_4_-Na_2_HPO_4_, pH 7.3, 2 mM K_3_Fe(CN)_6_, 2 mM K_4_Fe(CN)_6_, 1 mM 5-bromo-4-chloro-3-indolyl-β-D-glucuronide (X-Gluc), 0.1% (v/v) Triton X-100) at 37 °C for 6~16 h. Then the stained leaves were rinsed with 95% ethanol (at least 3 times, each time 15 min) in a high temperature (above 90 °C) to remove chlorophylls from plant tissues until the leaves were totally cleared and photographed.

### RNA extraction and real time RT-PCR assay

For the total RNA extraction, the *C. intermedia* leaves injected with *A. tumefaciens* transformed with pCanG-HA empty vector or *CiDREB1C* (GenBank: MG748598) were harvested at various time points, including 0, 2, 3, 4, 5, 6, 7, 8, 9, 12, 15, 18, 21, 24 and 27 days after infiltration. Total RNA was isolated from each sample according to the manufactures’ instruction (Invitrogen) of TRIzol reagent. About 2 μg of total RNA was reverse-transcribed into cDNA with oligo (dT)18 primers according to TransScript gDNA Removal and cDNA Synthesis SuperMix Kit (TransGen, Beijing, China, Cat# AT311). For real-time PCR analysis, the cDNA was diluted 16-fold with sterile DEPC water and 5 μL was added into a 20 μL PCR reaction. The real-time PCR was performed using SYBR Green I Master (Roche) on a LightCycler 480 system (Roche, Basel, Switzerland), which the following cycling parameters: 95 °C for 30 s, followed by 40 cycles of 95 °C for 5 s, 60 °C for 30 s and 72 °C for 15 s. A melting curve was generated for each sample at the end of running to access the purity of amplified products. *CiEF1α* (GenBank: KC679842) gene was used as internal references to normalize the samples. The expression level was calculated from the cycle threshold based on the 2^-ΔΔCT^ and 2^-ΔCT^ methods, with three technical replicates were performed at each experiment and at least two independent repetitions of the biological experiments were performed. The primers used in this study were listed in Additional file 5: Table S1.

### Stress resistance detection of transiently transformed in *C. intermedia*

Drought tolerance was detected mainly according to Wan et al. [40], and with slightly modification. Plants were grown in soil for about 20 days with sufficient watering. Then, the plant leaves were infiltrated with *A. tumefaciens* containing pCanG-HA empty vector or *CiDREB1C* gene. After infiltrated for 2 days, the plants were subjected to drought treatment by halting irrigation, and the plants infiltrated with pCanG-HA empty vector were used as control. Every pot was placed under the same conditions and the position of each pot was changed randomly every day. When the seedlings showed obvious wilting, yellowing and lethal effects of dehydration, the watering was restored. The survival rate of each line was calculated and the total chlorophyll content was measured after 8 days of re-watering. In the above experiment, there was four plants in each pot and at least four pots for both control and transient expression *CiDREB1C* seedlings. The photographs were taken immediately for at 17 days after drought treatments and at 4 days or 8 days after re-watering. The experiments were carried out with three biological replicates.

For salt stress, we mainly referred to Han et al. [41] and Wan et al. [42], and with minor modifications. Briefly, after infiltrated for 3 days, the plants were irrigated with 250 mmol/L NaCl solution and those infiltrated with pCanG-HA empty vector were taken as control. We poured about 2~3 L NaCl solution into the big tray containing pots with seedlings, which allowed each pot to take up enough NaCl solution from bottom to top naturally, and then poured out the excess NaCl solution after 24 h soaking. The NaCl solution was watered every 4~5 days until the seedlings showed apparent phenotype, the survival rate of each line was calculated, and the total chlorophyll content and seedlings growth rate were measured. The seedlings growth rate is the ratio of the height of seedlings growth during treatment to the total height of seedlings, which could reflect growth status and growth rate of seedlings under different stresses. The photographs were taken immediately at 11 and 21 days respectively after NaCl treatments. The experiments were carried out with three biological replicates.

For ABA treatment, the plants were sprayed with ABA aqueous solution (100 μmol/L, with 0.01% Silwet L-77) and the plants infiltrated with pCanG-HA empty vector were taken as control. We spray ABA solution on the leaves of each plant until the surface of leaves were all covered with ABA solution. The ABA aqueous solution was sprayed 4~5 times a day until the seedlings leaves showed obvious detachment and wilting phenotype, the survival rate and leaf drop rates of each line was calculated, and the total chlorophyll content was measured. The photographs were taken immediately at 4 or 10 days after ABA treatments. The experiments were also carried out with three biological replicates.

## Additional files

Additional file 1: **Figure S1.** Demonstration of *C. intermedia* leaves infected by syringe injection. a. Twenty-day old *C. intermedia* seedlings before injection. b. Hand-injection of the adaxial side of leaves. c. Leaves before (up panel) and after injection (lower panel) (DOCX 663 kb)

Additional file 2: **Figure S2.** Drought resistance detection of *C. intermedia* seedlings transiently expressing *CiDREB1C* a. Orthographic view of transient expression of *CiDREB1C* gene in drought resistance detection b. Oblique view of transient expression of *CiDREB1C* gene in drought resistance detection. Days after infiltration was indicated in parentheses. Bar = 2 cm *n* = 16 C: control (DOCX 902 kb)

Additional file 3: **Figure S3.** Salt tolerance detection of *C. intermedia* seedlings transiently expressing *CiDREB1C* a. Orthographic view of transient expression of *CiDREB1C* gene in salt tolerance detection b. Oblique view of transient expression of *CiDREB1C* gene in salt tolerance detection. Days after infiltration was indicated in parentheses. Bar = 2 cm n = 16 C: control (DOCX 855 kb)

Additional file 4: **Figure S4.** ABA tolerance detection of *C. intermedia* seedlings transiently expressing *CiDREB1C* a**.** Orthographic view of transient expression of *CiDREB1C* gene in ABA tolerance detection b. Oblique view of transient expression of *CiDREB1C* gene in ABA tolerance detection. Days after infiltration was indicated in parentheses. Bar = 2 cm n = 16 C: control (DOCX 2501 kb)

Additional file 5: **Table S1.** Primers used for qRT-PCR analysis in this work. F, forward primer; R, reverse primer (DOCX 14 kb)

## Abbreviations

ABA: Abscisic acid
carb: Carbenicillin resistance
*Ci*: *Caragana intermedia*
*CORs*: *Cold-regulated*
DEPC: Diethyl pyrocarbonate
DREB: Dehydration Responsive Element Binding protein
*EF1α*: *Elongation factor 1α*
gent: Gentamycin resistance
GUS: β-glucuronidase
kan: Kanamycin resistance
*RDs*: *Response to desiccation*
rif: Rifampicin resistance
rpm: Revolutions per minute
strep: Streptomycin resistance
X-Gluc: 5-bromo-4-chloro-3-indolyl-β-D-glucuronide
