# Supplementary figures and images for: Development of Agrobacterium-mediated transient expression system in Caragana intermedia and characterization of CiDREB1C in stress response
Source: BMC Plant Biol. 2019 Jun 6;19:237. doi: 10.1186/s12870-019-1800-4 (PMC6554893; doi:10.1186/s12870-019-1800-4)

**Additional file 1: Figure S1**

**
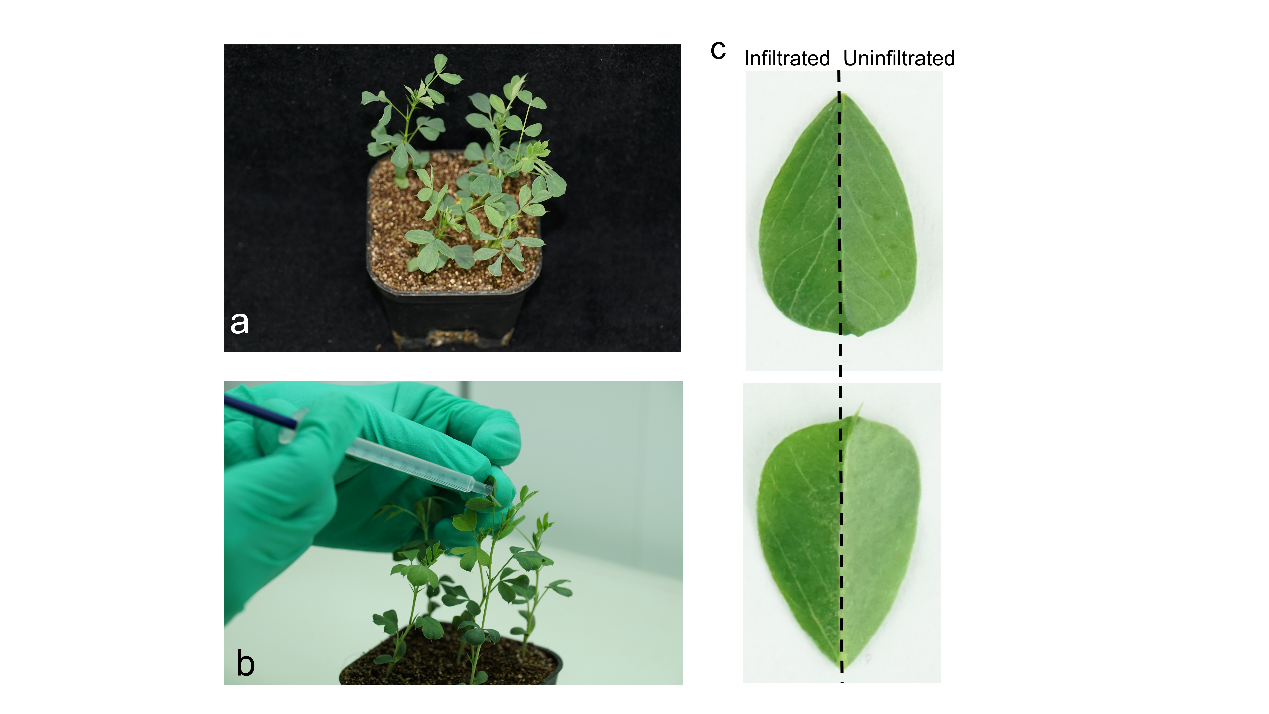
**

Supplement: Supplementary file 1 — Figure S1. Demonstration of C. intermedia leaves infected by syringe injection. a. Twenty-day old C. intermedia seedlings before injection. b. Hand-injection of the adaxial side of leaves. c. Leaves before (up panel) and after injection (lower panel) (DOCX 663 kb) [file 12870_2019_1800_MOESM1_ESM.docx]

**Additional file 2: Figure S2**


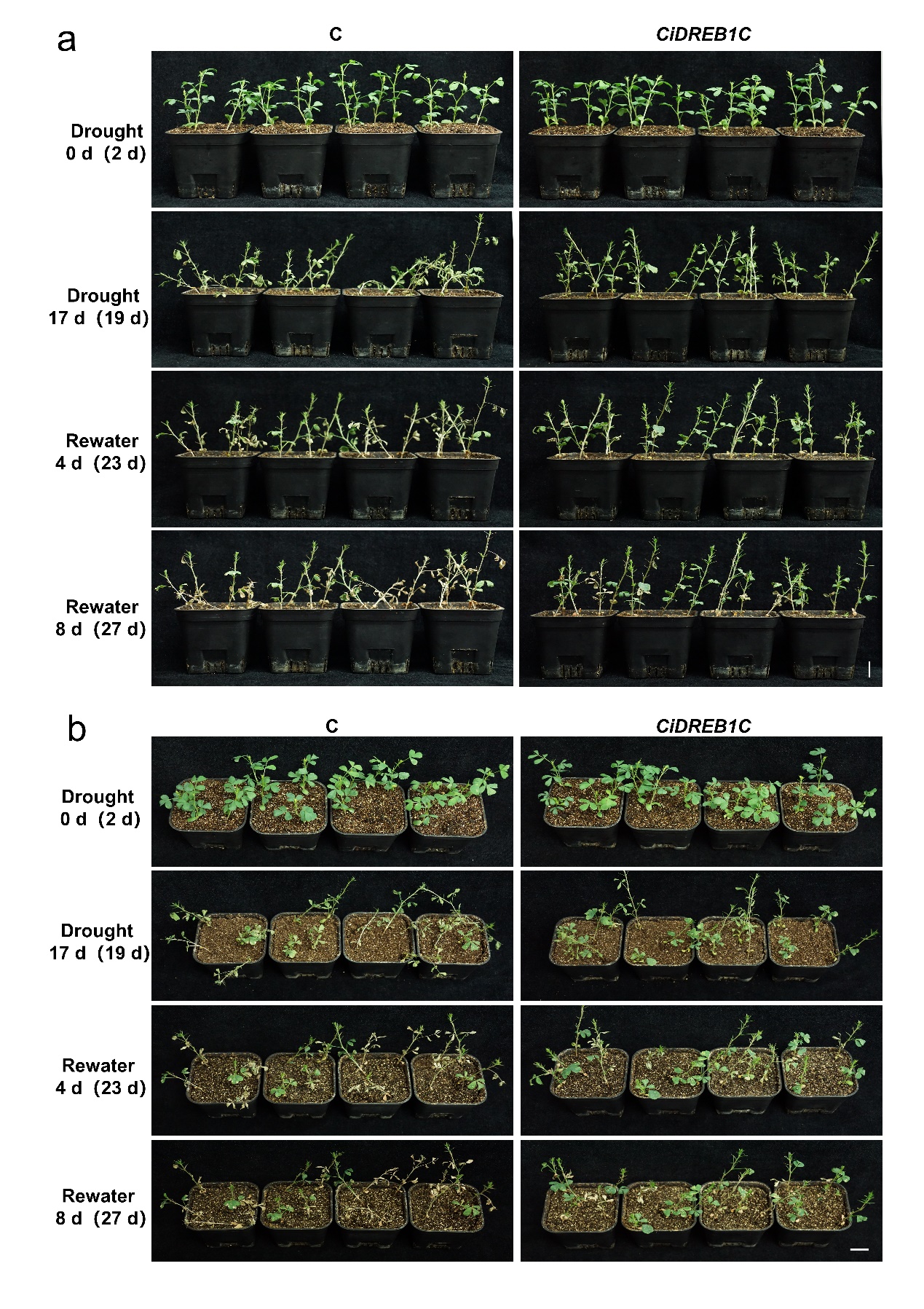

Supplement: Supplementary file 2 — Figure S2. Drought resistance detection of C. intermedia seedlings transiently expressing CiDREB1C a. Orthographic view of transient expression of CiDREB1C gene in drought resistance detection b. Oblique view of transient expression of CiDREB1C gene in drought resistance detection. Days after infiltration was indicated in parentheses. Bar = 2 cm n = 16 C: control (DOCX 902 kb) [file 12870_2019_1800_MOESM2_ESM.docx]

**Additional file 3: Figure S3**


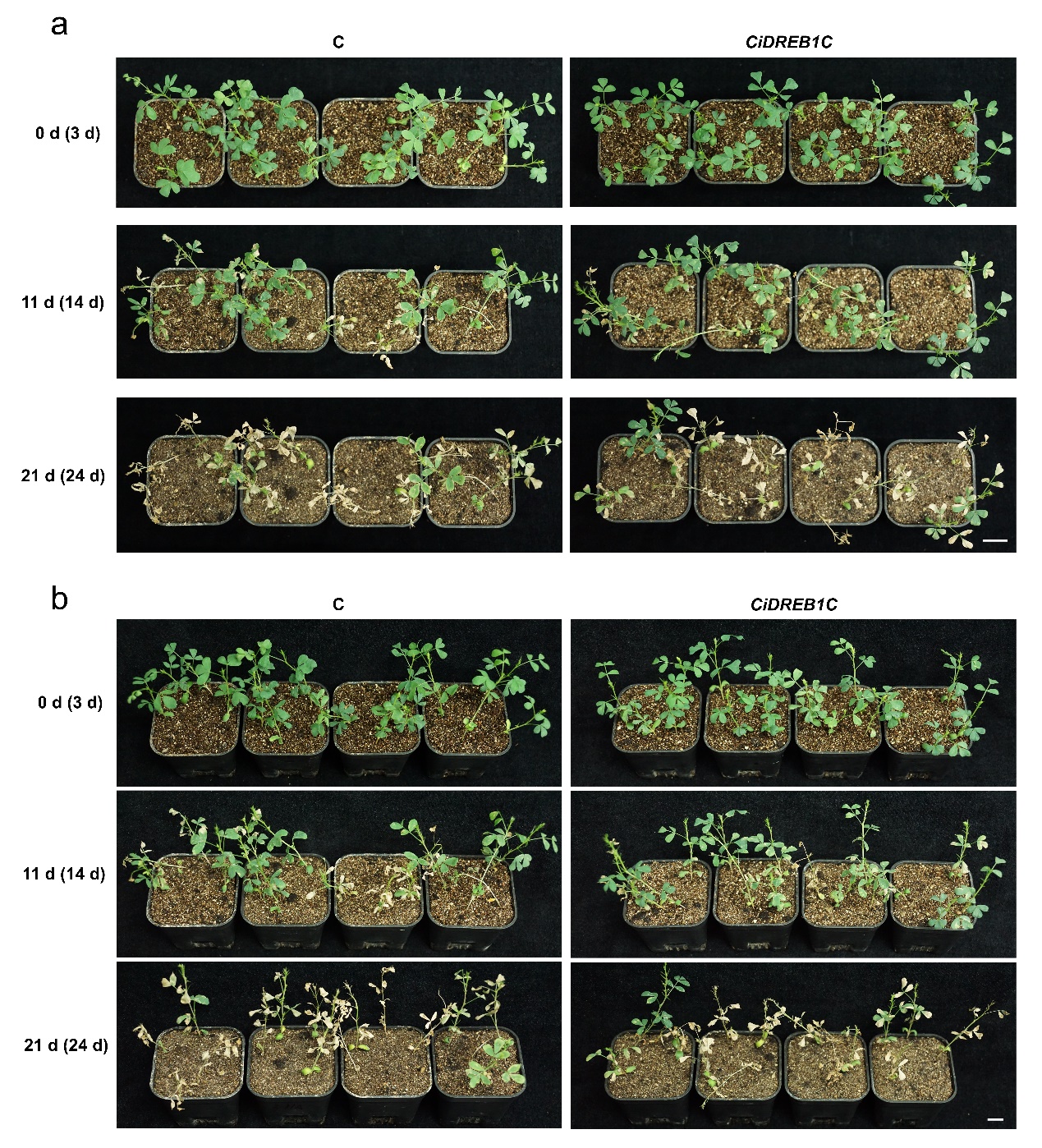

Supplement: Supplementary file 3 — Figure S3. Salt tolerance detection of C. intermedia seedlings transiently expressing CiDREB1C a. Orthographic view of transient expression of CiDREB1C gene in salt tolerance detection b. Oblique view of transient expression of CiDREB1C gene in salt tolerance detection. Days after infiltration was indicated in parentheses. Bar = 2 cm n = 16 C: control (DOCX 855 kb) [file 12870_2019_1800_MOESM3_ESM.docx]

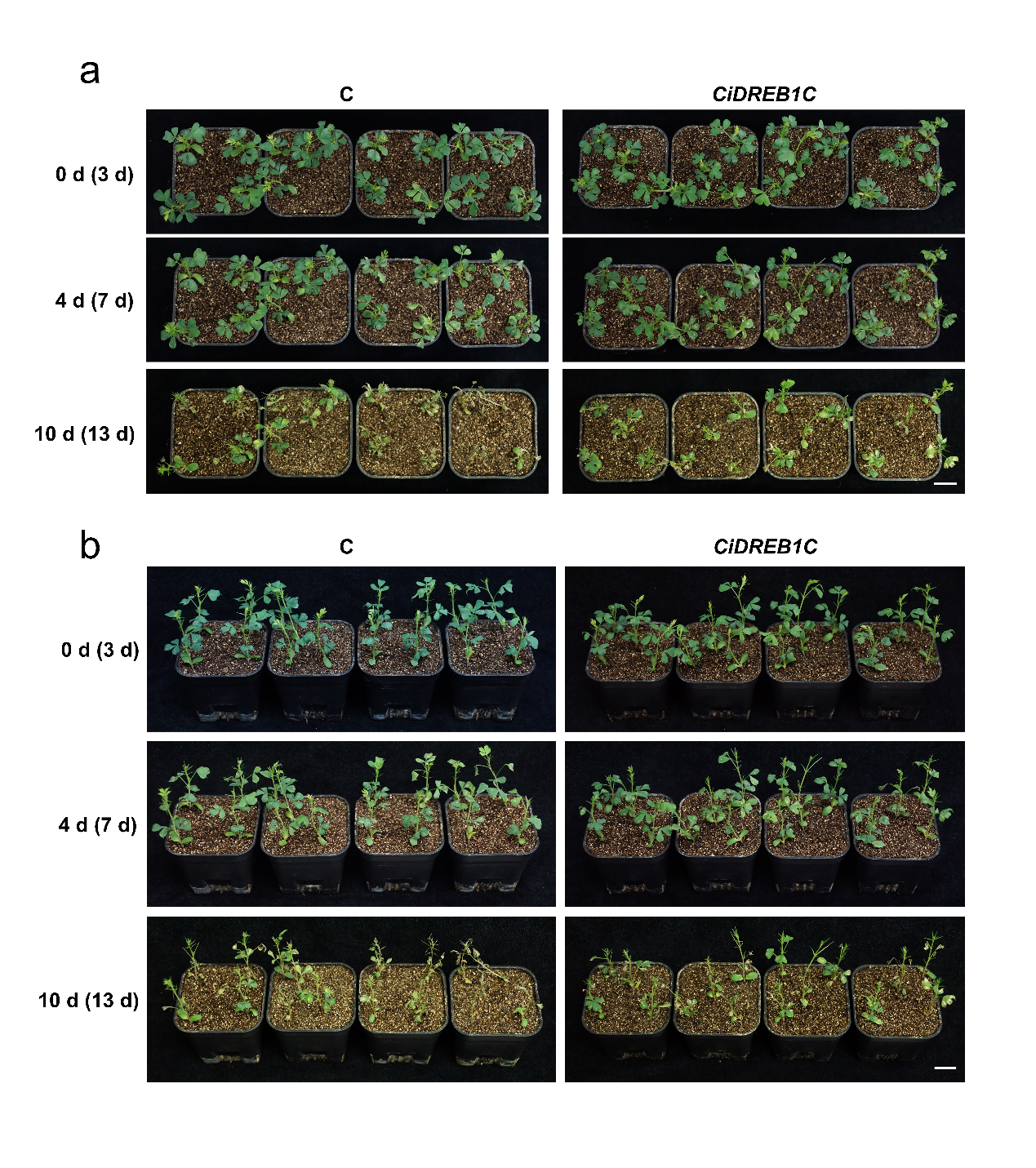
**Additional file 4: Figure S4**

Supplement: Supplementary file 4 — Figure S4. ABA tolerance detection of C. intermedia seedlings transiently expressing CiDREB1C a. Orthographic view of transient expression of CiDREB1C gene in ABA tolerance detection b. Oblique view of transient expression of CiDREB1C gene in ABA tolerance detection. Days after infiltration was indicated in parentheses. Bar = 2 cm n = 16 C: control (DOCX 2501 kb) [file 12870_2019_1800_MOESM4_ESM.docx]
