# Supplementary material for: Development of Agrobacterium-mediated transient expression system in Caragana intermedia and characterization of CiDREB1C in stress response
Source: BMC Plant Biol. 2019 Jun 6;19:237. doi: 10.1186/s12870-019-1800-4 (PMC6554893; doi:10.1186/s12870-019-1800-4)
Supplement: Supplementary file 5 — Table S1. Primers used for qRT-PCR analysis in this work. F, forward primer; R, reverse primer (DOCX 14 kb) [file 12870_2019_1800_MOESM5_ESM.docx]

**Additional file 5: Table S1**

| Genes | Polarity | Primer sequences (5'to3') | Usage |
| --- | --- | --- | --- |
| *CiEF1a* | F | TGGGTGGGACATTCTCTGATT | qRT-PCR |
| *CiEF1a* | R | GCACGGTTCACTTCTTCTTAGC | qRT-PCR |
| *CiDREB1C* | F | TCTGGCTTGGAACATTTTCTAACC | qRT-PCR |
| *CiDREB1C* | R | CCTCACAATCTCCTCCTCACTGG | qRT-PCR |
